# Genome-Wide Association Meta-Analysis of Cortical Bone Mineral Density Unravels Allelic Heterogeneity at the *RANKL* Locus and Potential Pleiotropic Effects on Bone

**DOI:** 10.1371/journal.pgen.1001217

**Published:** 2010-11-18

**Authors:** Lavinia Paternoster, Mattias Lorentzon, Liesbeth Vandenput, Magnus K. Karlsson, Östen Ljunggren, Andreas Kindmark, Dan Mellstrom, John P. Kemp, Caroline E. Jarett, Jeff M. P. Holly, Adrian Sayers, Beate St. Pourcain, Nicholas J. Timpson, Panos Deloukas, George Davey Smith, Susan M. Ring, David M. Evans, Jon H. Tobias, Claes Ohlsson

**Affiliations:** 1 Medical Research Council Centre for Causal Analyses in Translational Epidemiology, University of Bristol, Bristol, United Kingdom; 2 School of Social and Community Medicine, University of Bristol, Bristol, United Kingdom; 3 Center for Bone and Arthritis Research, Departments of Internal Medicine and Geriatrics, Sahlgrenska Academy, University of Gothenburg, Gothenburg, Sweden; 4 Clinical and Molecular Osteoporosis Research Unit, Department of Clinical Sciences, Lund University, Malmö, Sweden; 5 Department of Orthopaedics, Skåne University Hospital, Malmö, Sweden; 6 Department of Medical Sciences, University Hospital, Uppsala, Sweden; 7 Department of Clinical Science at North Bristol, University of Bristol, Bristol, United Kingdom; 8 Wellcome Trust Sanger Institute, Cambridge, United Kingdom; Georgia Institute of Technology, United States of America

## Abstract

Previous genome-wide association (GWA) studies have identified SNPs associated with areal bone mineral density (aBMD). However, this measure is influenced by several different skeletal parameters, such as periosteal expansion, cortical bone mineral density (BMD_C_) cortical thickness, trabecular number, and trabecular thickness, which may be under distinct biological and genetic control. We have carried out a GWA and replication study of BMD_C_, as measured by peripheral quantitative computed tomography (pQCT), a more homogenous and valid measure of actual volumetric bone density. After initial GWA meta-analysis of two cohorts (ALSPAC n = 999, aged ∼15 years and GOOD n = 935, aged ∼19 years), we attempted to replicate the BMD_C_ associations that had p<1×10^−5^ in an independent sample of ALSPAC children (n = 2803) and in a cohort of elderly men (MrOS Sweden, n = 1052). The rs1021188 SNP (near *RANKL*) was associated with BMD_C_ in all cohorts (overall p = 2×10^−14^, n = 5739). Each minor allele was associated with a decrease in BMD_C_ of ∼0.14SD. There was also evidence for an interaction between this variant and sex (p = 0.01), with a stronger effect in males than females (at age 15, males −6.77mg/cm^3^ per C allele, p = 2×10^−6^; females −2.79 mg/cm^3^ per C allele, p = 0.004). Furthermore, in a preliminary analysis, the rs1021188 minor C allele was associated with higher circulating levels of sRANKL (p<0.005). We show this variant to be independent from the previously aBMD associated SNP (rs9594738) and possibly from a third variant in the same RANKL region, which demonstrates important allelic heterogeneity at this locus. Associations with skeletal parameters reflecting bone dimensions were either not found or were much less pronounced. This finding implicates *RANKL* as a locus containing variation associated with volumetric bone density and provides further insight into the mechanism by which the *RANK*/*RANKL*/*OPG* pathway may be involved in skeletal development.

## Author Summary

Previous studies that have identified genetic polymorphisms involved in bone density have used a technique that cannot differentiate between cortical and trabecular bone. We have carried out the first genome-wide association study using a bone scanning method that can differentiate between the constituent parts of bone. We found a genetic variant (rs1021188) near the *RANKL* gene that was associated with the density of cortical bone in the three cohorts that we studied (ranging in age from 15 to 78 years old). We also found that this variant may have a more prominent effect on cortical bone density in males than females. In addition, the minor C allele of rs1021188 was associated with higher circulating levels of free RANKL. Although the *RANKL* gene has been previously identified as being important for bone structure (albeit with a different SNP showing association), we show for the first time that this may be primarily due to its influence on the density of cortical bone, rather than the size of the bone or other bone features.

## Introduction

Genome-wide association studies have identified reliable genetic associations with Dual x-ray absorptiometry (DXA)-derived measures related to bone mass, such as areal bone mineral density (aBMD) [1]–[4]. aBMD, is a commonly used skeletal trait measure on the basis of its ability to predict fractures at clinically important sites [5]. However, this measurement is influenced by several different skeletal parameters such as periosteal expansion, cortical BMD (BMD_C_), cortical thickness, trabecular number and trabecular thickness [6], all measures which may be under distinct systems of biological and genetic control. Currently, it is unclear which of these more precise bone related phenotypes identified genetic associates are associated with, whether work on aBMD is telling us more about bone size or growth [7], or what genetic variation has been missed through the use of more global bone measures.

**Figure 1:**
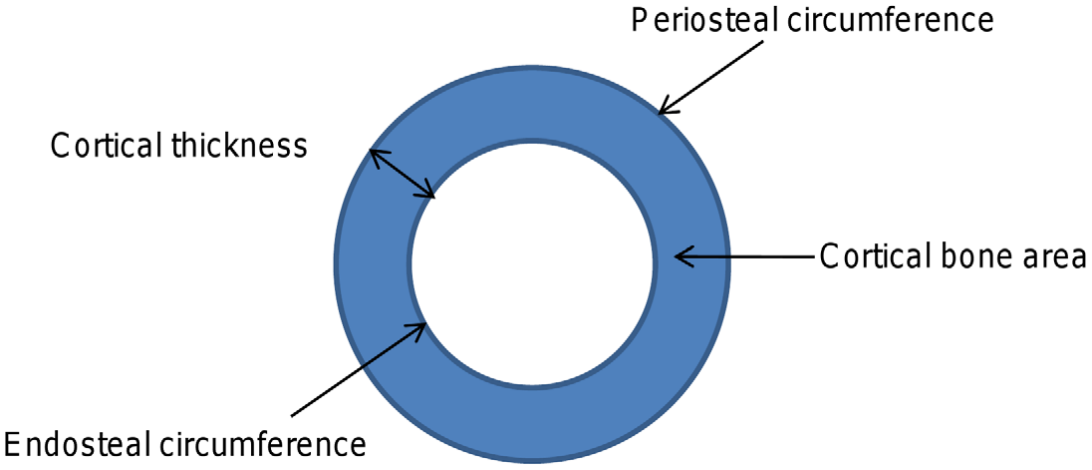
Cartoon depicting different cortical dimensions derived from tibial pQCT scans. Cortical bone mass is derived from the product of cortical bone area and cortical density.

Devices such as peripheral quantitative computed tomography (pQCT), which measure cross sections of predominantly cortical or trabecular bone, enable the different constituents of bone mass to be analysed separately (Figure 1) and may offer advantages over DXA in terms of identifying genetic correlates of specific bone phenotypes. Recently we examined whether genetic polymorphisms found to be associated with aBMD in recent GWA studies were also related to pQCT parameters, based on analysis of adolescents from the Avon Longitudinal Study of Parents and Children (ALSPAC) and young adult men from the ‘Gothenburg Osteoporosis and Obesity Determinants’ (GOOD) cohort [8]. We found that rs3018362 (near *RANK*), and rs4355801 and rs6993813 (near *OPG*), reported as being associated with aBMD in previous GWA studies [1], [3], were associated with BMD_C_ as measured by tibial pQCT, but not with any other pQCT phenotype. This raised the possibility that BMD_C_ is a potentially useful phenotype for detecting novel genetic influences on the skeleton, possibly reflecting the fact that this measure is entirely size independent.

Here we perform a GWA study of BMD_C_, based on data from tibial pQCT scans in the ALSPAC and GOOD cohorts. We present the results of our initial GWA meta-analysis, along with those from replication studies involving a further set of individuals from ALSPAC, and the MrOS Sweden cohort of elderly men.

## Results

Table 1 displays the means and standard deviations of bone traits for the four cohorts (ALSPAC discovery, GOOD discovery, ALSPAC replication and MrOS Sweden replication). Overall aBMD and BMDc were higher in the young adult men in the GOOD cohort when compared to the younger subjects in the ALSPAC cohort and the older men of MrOS Sweden. BMD_C_ is positively correlated with cortical thickness and trabecular BMD, but inversely correlated with the other pQCT traits, demonstrating the complicated interaction between these traits (Table 2). There is much lower correlation between BMD_C_ and BMD as measured by DXA (irrespective of measurement site).

**Table 1:** Characteristics of the included cohorts.

	ALSPAC (discovery)		ALSPAC (replication)		GOOD (discovery)		MrOS Sweden (replication)	
	n = 999		n = 2803		n = 935		n = 1052	
Age, years	15.4	(0.22)	15.5	(0.29)	18.9	(0.6)	78.7	(3.0)
Men, no. (%)	466	(47%)	1349	(48%)	935	(100%)	1052	(100%)
Height, cm	169.5	(8.2)	169.2	(8.3)	181.6	(6.6)	173.9	(6.4)
Weight, kg	61.0	(10.7)	61.1	(11.4)	73.9	(11.6)	79.2	(11.2)
Position of cortical section from distal end of tibia	50%		50%		25%		38%	
cortical BA, mm^2^	300.5	(48.3)	300.4	(50.3)	270.4	(34.5)	324.9	(46.7)
cortical BMC, mg	330.0	(50.5)	330.1	(52.9)	312.5	(39.5)	367.1	(56.1)
cortical BMD, mg/cm^3^	1100.0	(38.1)	1100.6	(38.8)	1155.7	(19.9)	1127.7	(40.8)
cortical Th, mm	5.40	(0.65)	5.38	(0.69)	4.43	(0.51)	5.17	(0.75)
cortical PC, mm	72.6	(6.0)	72.7	(6.2)	75.0	(4.9)	79.6	(5.9)
cortical EC, mm	38.7	(5.8)	38.9	(6.0)	47.2	(5.5)	47.1	(7.9)
Position of trabecular section from distal end of tibia	NA		NA		4%		4%	
trabecular BMD, mg/cm^3^	NA		NA		265.6	(33.9)	217.4	(37.5)
Total body BMD, g/cm^2^	n = 4003	1.03	(0.09)		1.25	(0.10)	NA	
Femoral neck BMD, g/cm^2^	n = 3328	0.98	(0.12)		1.17	(0.16)	0.83	(0.13)
Lumbar spine BMD, g/cm^2^	NA				1.21	(0.15)	1.14	(0.20)

Values are mean(SD), unless otherwise stated.

BA = bone area, BMC = bone mineral content, BMD = bone mineral density, Th = thickness, PC = periosteal circumference, EC = endosteal circumference.

**Table 2:** Spearman's rank correlation coefficients (rho) between the bone traits in the GOOD cohort.

	pQCT phenotypes						DXA phenotypes		
	cort BMC	cort BA	cort PC	cort EC	cort Th	Trab BMD	TB BMD	FN BMD	LS BMD
cort BMD	−0.017	−0.140	−0.346	−0.369	0.109	0.112	0.069	0.039	0.074
cort BMC		0.991	0.678	0.144	0.790	0.502	0.785	0.676	0.608
cort BA			0.717	0.191	0.766	0.483	0.767	0.664	0.591
cort PC				0.796	0.144	0.132	0.500	0.405	0.387
cort EC					−0.439	−0.224	0.071	0.016	0.048
cort Th						0.584	0.642	0.587	0.498
trab BMD							0.643	0.650	0.534

BA = bone area, BMC = bone mineral content, BMD = bone mineral density, Th = thickness, PC = periosteal circumference, EC = endosteal circumference, TB = total body, FN = femoral neck, LS = lumbar spine.

**Figure 2:**
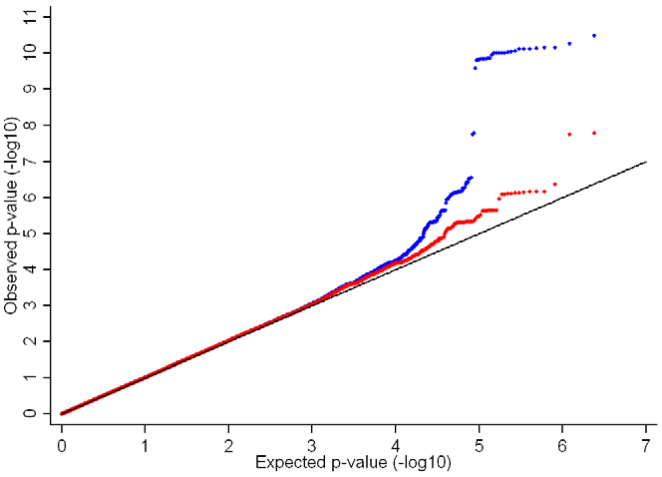
QQ plot of the ALSPAC and GOOD genome-wide meta-analysis of BMD_C_. Blue points show the full genome-wide results. Red points show the genome-wide results excluding the RANKL region (chr 13: 42000–42150Kb).

**Figure 3:**
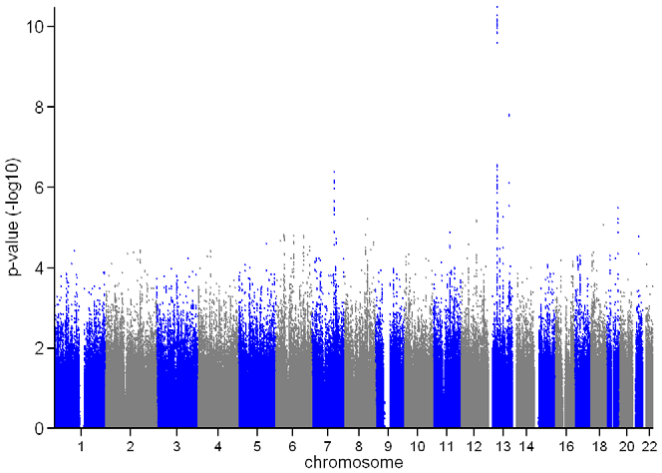
Manhattan plot of the ALSPAC and GOOD genome-wide meta-analysis of BMD_C_.

In the genome-wide association meta-analysis of the ALSPAC discovery cohort and GOOD cohort there was little systematic inflation of test statistics (λ_GC_ = 1.021 (1.0004 for ALSPAC; 1.010 for GOOD), but a marked deviation from the null amongst the lowest observed p-values (Figure 2). The greatest evidence for association between genetic variation and BMD_C_ was seen at rs1021188 (ALSPAC β = −7.63; GOOD β = −6.02, overall p = 3×10^−11^ (p = 4×10^−11^ after applying genomic control)) on chromosome 13, slightly upstream of the *RANKL* gene (Figure 3). We selected the nine regions with p<1×10^−5^ and carried out analyses conditional on the most associated SNP in that region to check if there were multiple independently associated SNPs in each region (A full list of SNPs that exhibit nominal evidence of association (p<1×10^−5^) with BMD_C_ can be found in the supplementary online material, Table S1). The *RANKL* region of chromosome 13 was the only region for which a second SNP (rs9525613) still showed marginal association (p = 0.008) when conditioning on the most associated SNP in this region (rs1021188). We selected both of these SNPs for replication follow-up, in addition to the top ranking SNP from each of the other regions (rs7338502, rs211804, rs8102334, rs17066364, rs9541712, rs16877095, rs4280044, rs11875173). As well as our primary analyses which adjusted for age, sex (ALSPAC only), height and weight(ln), we also carried out analyses adjusting for only age and sex and found broadly similar results.

In both the replication cohorts the *RANKL* SNP (rs1021188) was the only variant to be consistently associated and the only SNP to reach genome-wide significance in a p-value meta-analysis of the four cohorts (p = 2×10^−14^, total n = 5739) (Table 3). On carrying out an inverse-variance meta-analysis of the ALSPAC, GOOD and MrOS Sweden cohorts each minor C allele was associated with a clear decrease in BMD_C_ of ∼0.14 SD (Table 4), explaining 0.6%, 2.2% and 0.5% of the variation in the ALSPAC cohort, GOOD cohort and MrOS Sweden cohort, respectively.

**Table 3:** Top cortical BMD GWAS meta-analysis hits, with replication and meta-analysis results of all four cohorts.

				Discovery									Replication								Combined all cohorts	
				Alspac discovery				GOOD					Alspac replication				MrOS Sweden					
Gene	SNP	position	Effect allele	allele freq	N	beta (se)	p	allele freq	N	beta (se)	p	Meta-Analysis P-value	allele freq	N	beta (se)	p	allele freq	N	beta (se)	p	N	Meta-Analysis P-value
*RANKL*	rs1021188	13:42014133	C	0.18	999	−7.63 (1.67)	5E-06	0.15	935	−6.02 (1.24)	1E-06	3E-11	0.18	2753	−3.89 (1.01)	1E-04	0.15	1052	−5.97 (2.23)	7E-03	5739	2E-14
*RANKL*	rs9525613	13:41816648	T	0.30	999	−4.00 (1.34)	3E-03	0.33	935	−3.14 (0.95)	1E-03	9E-06	0.28	2784	−2.48 (0.85)	0.014	0.30	1046	−1.80 (1.74)	0.300	5764	2E-06
*STK24*	rs7338502	13:97948011	T	0.05	999	−12.05 (2.88)	3E-05	0.04	935	−8.34 (2.20)	2E-04	2E-08	0.05	2789	2.12 (1.77)	0.231	0.04	1043	−0.92 (3.86)	0.812	5766	0.011
*IMMPL2*	rs211804	7:109878696	A	0.18	999	6.35 (1.64)	1E-04	0.16	935	3.94 (1.21)	1E-03	4E-07	0.17	2771	1.33 (1.06)	0.212	0.16	1047	−4.13 (2.19)	0.060	5752	0.003
*ETFB*	rs8102334	19:56543541	G	0.51	999	−3.70 (1.28)	4E-03	0.47	935	−3.39 (0.92)	2E-04	3E-06	0.49	2784	0.41 (0.77)	0.589	0.50	1044	0.31 (1.62)	0.850	5762	0.025
*NUFIP1*	rs17066364	13:44413989	C	0.02	999	−11.56 (4.75)	0.015	0.03	935	−11.53 (1.03)	5E-05	4E-06	0.02	2764	0.80 (2.81)	0.775	0.03	1053	−1.62 (4.65)	0.728	5751	0.009
*-*	rs9541712	13:68481740	A	0.33	999	2.92 (1.33)	0.027	0.34	935	4.18 (0.98)	2E-05	6E-06			Failed		0.35	1013	−1.30 (1.72)	0.450	2947	0.001
*RSPO2*	rs16877095	8:109123844	T	0.16	999	6.04 (1.70)	4E-04	0.17	935	3.22 (1.14)	5E-03	6E-06	0.15	2789	0.20 (1.09)	0.854	0.16	1046	−3.33 (2.13)	0.118	5769	0.038
*E2F7*	rs4280044	12:76117369	G*	0.24	999	−5.95 (1.50)	7E-05	0.27	935	−2.42 (1.02)	0.018	7E-06	0.26	2767	1.30 (0.90)	0.148	0.27	1022	−1.96 (1.84)	0.284	5723	0.039
-	rs11875173	18:63006929	C	0.35	999	3.79 (1.44)	9E-03	0.32	935	3.80 (1.03)	2E-04	9E-06	0.32	2767	1.49 (0.84)	0.077	0.29	1028	0.33 (1.78)	0.854	5729	1E-04

* = Was in MrOS Sweden replaced with proxy SNP rs2369599 (Effect allele G).

Models adjusted for age, height and lnweight (and sex in ALSPAC).

Betas are mg/cm^3^ per effect allele.

**Table 4:** rs1021188 associations with bone parameters at different ages and meta-analyses results (combining ALSPAC, GOOD 19, and MrOS Sweden).

	ALSPAC 15 years (13 for FN BMD)					GOOD 19 years					GOOD 24 years					MrOS Sweden 78 years					Combined*		
*pQCT*	N	beta†	p	Beta and SE in SD		N	beta†	p	Beta and SE in SD		N	beta†	p	Beta and SE in SD		N	beta†	p	Beta and SE in SD		N	p	Beta, in SD
Cort BMD, mg/cm^3^	3729	−4.81	3E-08	**−0.13**	**0.02**	935	−6.02	1E-06	**−0.30**	**0.06**	729	−4.84	3E-04	−0.25	0.07	1052	−5.97	7E-03	**−0.15**	**0.06**	5716	8E-14	−0.14
Cort BMC, mg	3729	−2.21	0.059	**−0.04**	**0.02**	935	−1.85	0.382	**−0.05**	**0.05**	729	−2.81	0.284	−0.07	0.06	1052	−6.40	0.039	**−0.11**	**0.06**	5716	0.007	−0.05
Cort BA, mm^2^	3729	−0.63	0.548	**−0.01**	**0.02**	935	−0.26	0.888	**−0.01**	**0.05**	729	−1.26	0.581	−0.03	0.06	1052	−4.16	0.108	**−0.09**	**0.06**	5716	0.256	−0.02
Cort PC, mm	3729	0.27	0.035	**0.04**	**0.02**	935	0.32	0.191	**0.07**	**0.05**	729	0.17	0.545	0.04	0.06	1052	0.47	0.150	**0.08**	**0.06**	5716	0.005	0.05
Cort EC, mm	3729	0.60	3E-04	**0.10**	**0.03**	935	0.54	0.089	**0.10**	**0.06**	729	0.45	0.205	0.08	0.07	1052	1.19	0.011	**0.15**	**0.06**	5716	3E-06	0.11
Cort Th, mm	3729	−0.05	4E-03	**−0.08**	**0.03**	935	−0.03	0.279	**−0.07**	**0.06**	729	−0.05	0.250	−0.08	0.07	1052	−0.12	8E-03	**−0.16**	**0.04**	5716	1E-05	−0.09
Trab BMD, mg/cm^3^						934	−1.98	0.346	**−0.06**	**0.06**	728	−2.01	0.433	−0.06	0.07	1068	−3.81	0.080	**−0.10**	**0.06**	2002	0.055	−0.08

*Combined p-value and effect size in SD calculated using meta-analyses of ALSPAC, GOOD 19 years and MrOS Sweden (bold columns).

**†:** raw betas are in units per C allele. Models adjusted for age, height and lnweight (and sex in ALSPAC). GOOD 24 years sample is a sub-set of GOOD 19 years.

BA = bone area, BMC = bone mineral content, BMD = bone mineral density, Th = thickness, PC = periosteal circumference, EC = endosteal circumference, TB = total body, FN = femoral neck, LS = lumbar spine.

The association between rs1021188 and BMD_C_ was stronger in males (β = −6.77, p = 2×10^−6^) than females (β = −2.79, p = 0.004) in the entire ALSPAC cohort (and testing for an interaction between sex and genotype gave p = 0.01) (Table 5). Both male-only cohorts showed similar effects for rs1021188 and BMD_C_ (β = −6.02 GOOD, β = −5.97 MrOS Sweden).

**Table 5:** rs1021188 associations with BMD_C_ in both ALSPAC datasets stratified by sex.

	DISCOVERY SET				REPLICATION SET				WHOLE COHORT			
Sex	N	Beta (se)	p	interaction p	N	Beta (se)	p	interaction p	N	Beta(se)	p	interaction p
BOTH	976	−7.63 (1.67)	5.0E-06	0.074	2753	−3.89 (1.01)	1.0E-04	0.060	3729	−4.81 (0.86)	3.0E-08	0.012
MALE	455	−9.77 (2.62)	2.2E-04		1325	−5.69 (1.66)	6.4E-04		1780	−6.77 (1.41)	1.6E-06	
FEMALE	521	−4.84 (1.93)	1.2E-02		1428	−2.01 (1.12)	7.4E-02		1949	−2.79 (0.97)	4.1E-03	

Betas are mg/cm^3^ per C allele.

After removal of the *RANKL* region (chr 13: 42000–42150Kb) from the main analysis, there was evidence for a marked reduction in the inflation of test statistics, however the existence of some departure from the expected null distribution suggested that further loci remain to be identified (Figure 2).

**Figure 4:**
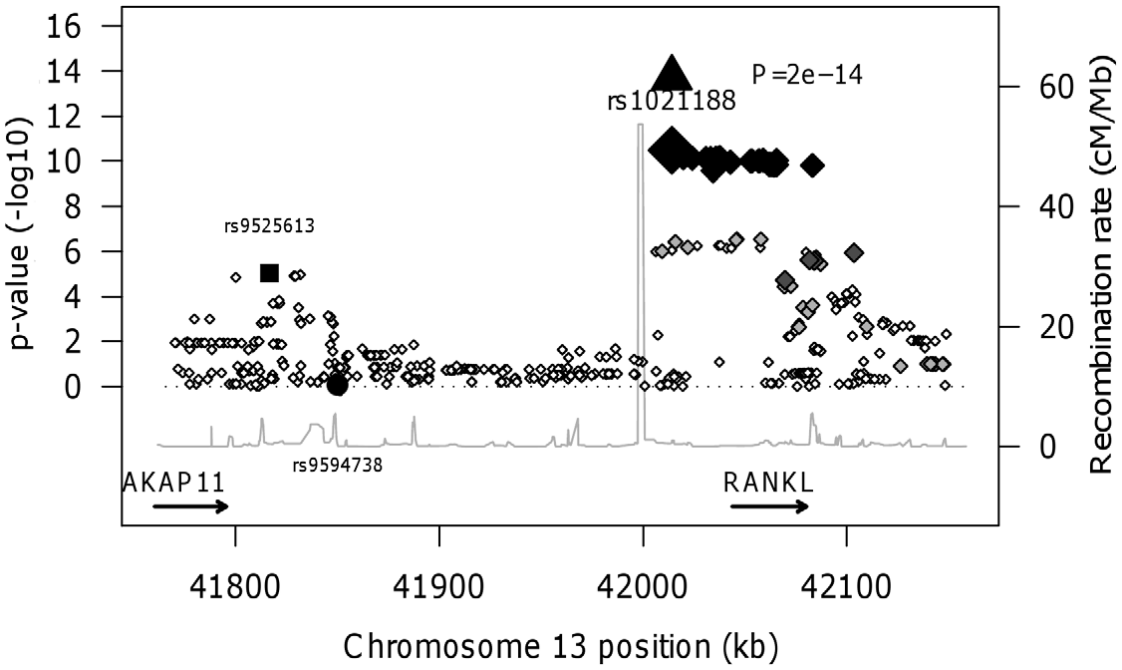
RANKL regional association plot of the ALSPAC and GOOD genome-wide meta-analysis of BMD_C_. Diamonds show the ALSPAC and GOOD GWA meta-analysis p-values, with darker shades indicating increasing linkage disequilibrium with rs1021188. The triangle shows the meta-analysis p-value of all cohorts (discovery and replication) for rs1021188. The black square shows the second SNP in this region which we attempted to replicate in this study (rs9525613) and the black circle shows the p-value of rs9594738, which has previously been associated with areal bone mineral density (3). The grey line shows the recombination rate across the region (data from HapMap).

We found no association between BMD_C_ and a RANKL variant (rs9594738) which has previously been associated with aBMD [2], [3]. We assessed the proximity of our most associated loci (rs1021188), the nearby rs9525613 (which we also attempted to replicate) and rs9594738 to the *RANKL* gene (Figure 4). We found that the rs1021188 SNP is much closer to the *RANKL* gene than the aBMD associated SNP (and rs9525613) and that there are high recombination rates between the two regions (r^2^ between the two SNPs is 0.01), indicating that these may be separate signals (Figure 4).

To determine if the association between rs1021188 and BMD_C_ was specific for this bone trait, we have tested for associations between the *RANKL* SNP (rs1021188) and other skeletal traits in ALSPAC, GOOD and MrOS Sweden. rs1021188 shows some evidence for association with endosteal circumference and cortical thickness, but to a much lesser extent than BMD_C_ and even less so for cortical content and periosteal circumference. Though the evidence for these associations comes mostly from the ALSPAC and MrOS cohorts, the effect sizes seen in the GOOD cohort are extremely consistent and the lack of association in this cohort is likely due to the reduced power found in this smaller study. Although there is some evidence for association between rs1021188 and total-body, femoral neck and lumbar-spine BMD (as measured by DXA), this appears to be weaker than the association seen with tibial cortical BMD, though with smaller sample sizes for these analyses these comparisons may not be robust (Table 4). In the ALSPAC cohort, the association between rs1021188 and BMD_C_ was only slightly attenuated when the analysis was adjusted for femoral neck BMD (β = −4.49, p = 5×10^−7^) or total body BMD (β = −3.64, p = 3×10^−5^), as measured by DXA.

**Figure 5:**
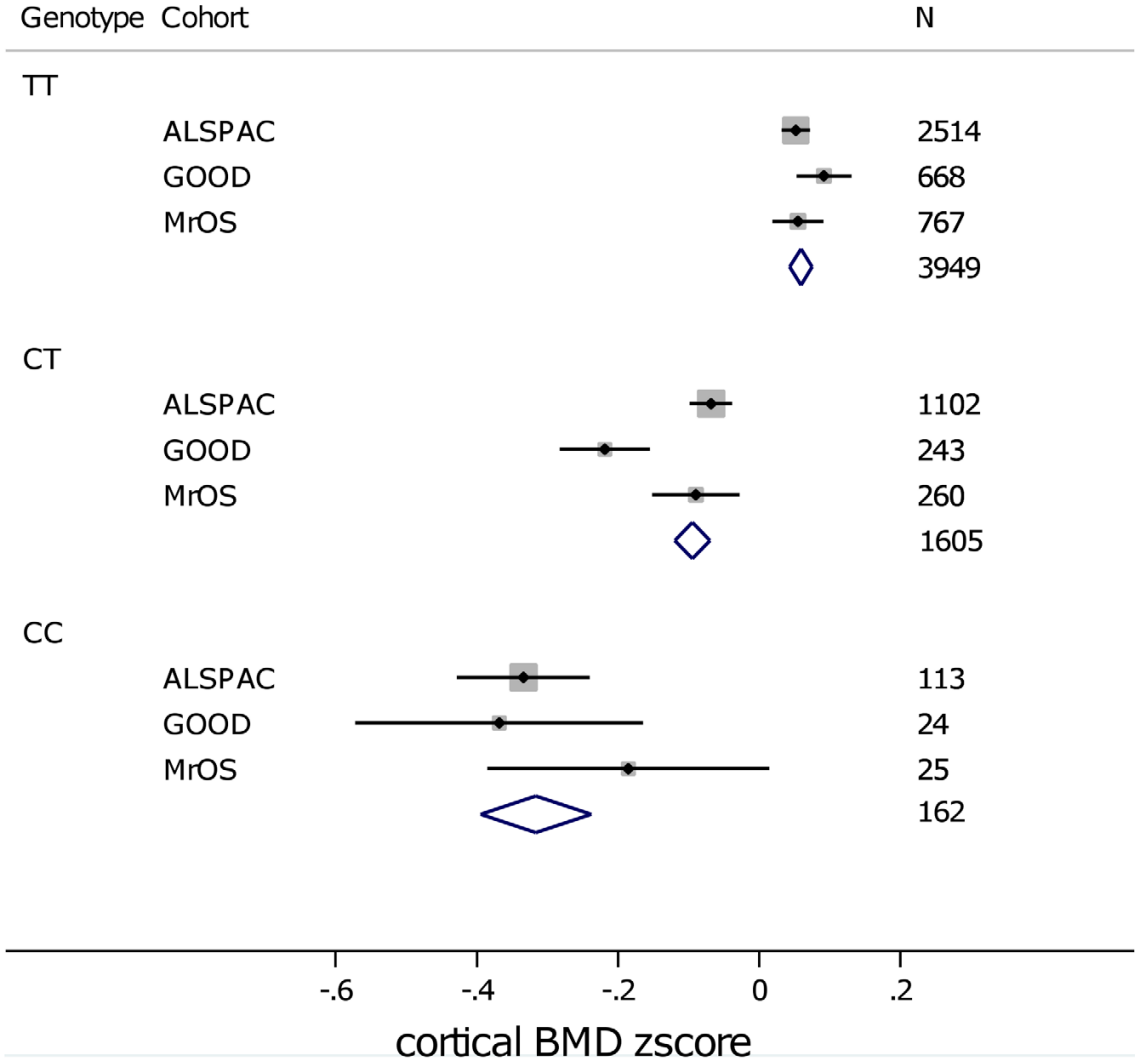
Mean (and standard error) BMD_C_ z-scores per rs1021188 genotype in each of the cohorts. Sample sizes are shown alongside each point. Diamonds show the combined z-score estimates per genotype (the width of the diamond represents the combined standard error).

In analysis of mean BMD_C_ z-scores per rs1021188 genotype in each of the cohorts rs1021188 had an approximately additive effect; with each C allele cortical BMD is decreased (Figure 5). This appears to be true across all ages, although the absolute BMD_C_ values vary.

**Figure 6:**
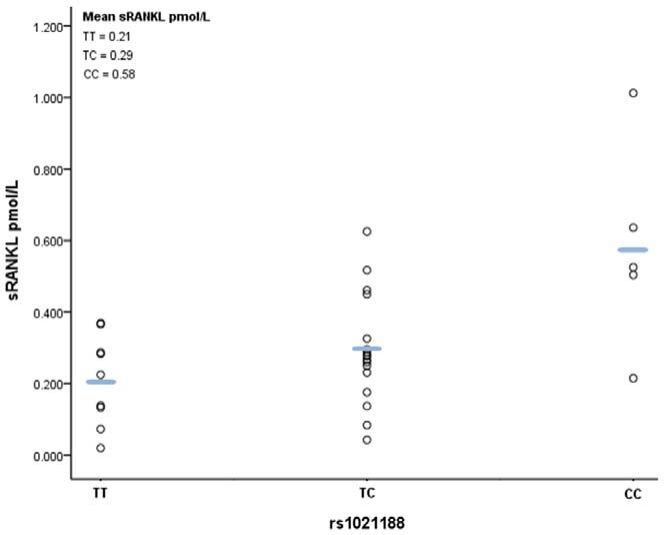
Scatter plot and mean values of free plasma RANKL levels according to rs1021188 genotype. Of the 37 subjects measured, replicate samples in six had a CV of >20% and were excluded, leaving 9, 17 and 5 samples with TT, CT and CC genotypes respectively (a single sample fell below the detection limit of the assay and was therefore entered at the lower detection value i.e 0.02pmol/l). A positive relationship was observed between number of minor rs1021188 alleles and plasma RANKL level (P = 0.005; linear regression analysis following Rank-Based Inverse Normal Transformation).

Finally, we analysed the relationship between rs1021188 genotype and plasma levels of soluble RANKL (sRANKL) in a small sample of male subjects from ALSPAC. A positive relationship was observed between number of minor rs1021188 alleles and sRANKL level (P = 0.005), such that sRANKL levels were over twice as great in those with CC versus TT genotypes (Figure 6).

## Discussion

In genomewide analysis of the specific bone phenotype BMD_C_, we have been able to identify genetic variants different to those found from equivalent studies based on aBMD. In particular, the rs1021188 SNP was found to be reliably associated with BMD_C_, but has not previously been reported to be associated with any other bone phenotype to date. rs1021188 is located ∼20Kb upstream of the receptor activator of nuclear factor-κB ligand gene (*RANKL*) on chromosome 13. The minor C allele was associated with a decrease in BMD_C_ (of ∼0.14SD, explaining 0.5–2.2%, of the variation - though the larger of these estimates is inflated by the more homogenous GOOD cohort having less overall variance) and there was also evidence for an interaction with sex, with larger effects being observed amongst males (though this study was not designed to test for sex-gene interactions). The effect of this marker on BMD_C_ was similar across all cohorts, suggesting that although our discovery set comprised adolescents/young adults, this marker influences cortical density to a similar extent in the elderly.

Whereas the rs1021188 (C allele) of *RANKL* showed a strong inverse association with BMD_C_, associations with cortical bone mass were somewhat weaker. Nevertheless, the size of the latter effect was still equivalent to that of a doubling of vigorous physical activity (as measured by accelerometer) in the ALSPAC cohort at the same age (unpublished). The weaker association between rs1021188 genotype and cortical bone mass compared with that of cortical density is likely to be explained by rs1021188 being unrelated to cortical bone size (reflected by cortical bone area), which is a major determinant of cortical bone mass. Although rs1021188 was not associated with cortical bone area as a whole, a relatively strong positive association was observed with endosteal circumference, whereas there was an equivalent inverse association with cortical thickness, together suggesting that rs1021188 increases endosteal expansion. There was also a weak positive association between this marker and periosteal circumference (possibly reflecting a compensatory response), which may explain why the increased endosteal expansion associated with this marker had little impact on overall cortical bone size. Since these analyses were adjusted for height, these findings imply that RANKL influences the rate of periosteal and endosteal expansion independently of vertical growth, consistent with a local action on cortical bone (see below).

The observation that the *RANKL* rs1021188 is associated with cortical BMD is consistent with our recent finding from a larger-scale candidate gene study in the ALSPAC and GOOD cohorts that other genes of the *RANK*/*RANKL*/*OPG* pathway are also associated with BMD_C_ [8]. Given the important role of this pathway in regulating osteoclast differentiation [9], these findings may reflect a response to enhanced net RANKL activity leading to greater osteoclast activation and hence stimulation of cortical remodeling. Our observation that the minor C allele of rs1021188 was associated with higher circulating levels of free RANKL in a preliminary analysis, as measured in a small number of ALSPAC subjects, is consistent with this interpretation. Increased bone resorption arising from higher RANKL levels is predicted to reduce BMD_C_ both by increasing cortical porosity and reducing the available time for secondary mineralization [10]. Increased bone resorption could also explain the relationship we observed between *RANKL* rs1021188 and increased endosteal resorption, which is also an osteoclast-dependent process.

Although the *RANKL* rs1021188 marker has not been reported to be associated with any bone-related trait before, a SNP (rs9594738) ∼190kb upstream of *RANKL* was strongly associated (standardized beta = −0.17, p = 2.0×10^−21^) with lumbar spine areal bone mineral density (aBMD) in a genome-wide association and replication study in four cohorts of mostly elderly subjects (with a high proportion of postmenopausal women) [3] and a perfect proxy SNP for rs9594738 (rs9533090, r^2^ = 1) was also strongly associated (standardized beta = −0.12, p = 5.4×10^−25^) with lumbar spine aBMD in a further meta-analysis of five genome-wide association studies of a wide age range (again with a high proportion of women) [2]. These studies also found these SNPs to be associated with hip aBMD, but to a lesser extent (rs9594738: standardized beta = −0.10, p = 1.9×10^−8^; rs9533090: standardized beta = −0.04, p = 3.9×10^−4^) and not with low trauma fractures (p = 0.23), demonstrating that there may be skeletal site heterogeneity in the associations with RANKL. In contrast, we have previously shown rs9594738 to not be associated with BMD_C_ in the ALSPAC and GOOD cohorts [8] and we show here that the two signals are likely to be independent. There are high recombination rates between the two regions where rs1021188 and rs9594738 are located, and so these two markers may well be tagging distinct functional *RANKL* variants which affect these different bone phenotypes individually. Alternatively, as rs9594738 is located ∼190kb upstream of *RANKL* and is in fact closer to another gene *AKAP11*, it may be that the two phenotypes are under the control of different genes entirely, although *AKAP11* has not to date been implicated in skeletal regulation. In addition another SNP (rs10507508) has also been shown to be associated (p = 0.0011) with aBMD after adjusting for 59 other SNPs in the region [3], possibly representing a further independent association with BMD at this locus. This allelic heterogeneity could be important for understanding the relationship between *RANKL* and bone phenotypes.

The differing genetic associations between aBMD and BMD_C_ (and site of investigation) at this *RANKL* locus may be explained by the genetic variants being associated with distinct properties of bone. Since analysis of lumbar spine BMD may be mostly assessing trabecular bone, previously identified *RANKL* variants may be particularly associated with this component. In contrast, the *RANKL* variant that we identify here may be more strongly associated with cortical bone, given that the tibial pQCT site (which formed the basis of this study) comprises solely of cortical bone. Here, total body BMD and femoral neck BMD showed only a weak association with the *RANKL* rs1021188 marker (and adjusting for these measures in the BMD_C_ analysis only slightly attenuated the association) and the lumbar spine BMD (which largely comprises trabecular bone) showed an association very similar to that seen for tibial trabecular BMD, implying that cortical associations may be missed using DXA to assess BMD. The implication that there is little relationship between cortical BMD and BMD as measured by DXA is consistent with the weak correlations we observed between cortical BMD and DXA measured BMD (irrespective of site). This is likely to reflect the fact that BMD as measured by DXA is mainly influenced by parameters such as overall bone size and cortical thickness, as opposed to the material density of bone and/or degree of cortical porosity (as reflected by pQCT measured cortical BMD). In contrast to BMD as measured by DXA which is related to clinical end points such as fracture risk, few if any studies have analysed cortical BMD measurements in relation to fracture risk. Nevertheless, recent findings have highlighted the importance of changes in determinants of cortical density, such as cortical porosity, in the pathogenesis of osteoporotic fracture [11].

Taken together with our previous report of associations between *RANK* and OPG variants and BMDc, our results provide further evidence that the *RANK*/*RANKL*/*OPG* axis affects the skeleton at least in part by influencing material density of cortical bone. Although the precise relationship between this phenotype and clinical end points such as fracture risk remains to be established, it is clear that the availability of precise and specific measures of bone health provide great resolution in the analysis of heritable contributions to this category of traits. It is also tempting to speculate that changes in BMD_C_ contribute to recent observations that the RANKL inhibitor denosumab reduces fracture risk [12]. Consistent with this possibility, administration of denosumab has been found to increase femoral BMD_C_ in mice with a knock-in of humanised RANKL [13].

In summary we have performed the first GWAS with cortical BMD. We identified a SNP (rs1021188) close to the *RANKL* gene with strong evidence for association with BMD_C_ in adolescent and young adult cohorts used for the discovery phase, as well as in our replication cohort of elderly men, suggesting this genetic effect acts over the whole lifetime. We also show some evidence for an interaction with sex for this variant, with the association being stronger in males. In addition, the minor C allele of rs1021188 was associated with higher circulating levels of free RANKL in a preliminary analysis. Consistent with an action of this variant via altered RANKL and hence osteoclast activity, an equivalent association was also observed with endosteal expansion as reflected by endosteal circumference. However, there was little relation between rs1021188 and overall cortical bone area, possibly reflecting compensatory changes in periosteal apposition. This signal appears to be independent of another previously reported association in this region between aBMD and rs9594738, demonstrating that there may be important allelic heterogeneity at this locus, and possibly indicating that these variants are associated with different components of bone structure. Further studies are justified to extend these findings, for example by performing further GWA studies with sufficient power to detect other important genetic influences on cortical BMD.

## Methods

### ALSPAC cohort

#### Participants

The Avon Longitudinal Study of Parents and their Children (ALSPAC) is a longitudinal population-based birth cohort that initially included over 13,000 women and their children in Avon, UK, in the early 1990s. This cohort is described in detail on the website (http://www.alspac.bris.ac.uk) and elsewhere [14]. Both mothers and children have been extensively followed from the 8th gestational week onwards using a combination of self-reported questionnaires, medical records and physical examinations. Ethical approval was obtained from the ALSPAC Law and Ethics committee and relevant local ethics committees, and written informed consent provided by all parents. Blood samples were taken and DNA extracted as previously described [15].

#### Bone measures

pQCT scans were performed on approximately 4500 children when they attended the age 15 research clinic at which time total body DXA scans were also performed. Results for hip DXA scans, collected at the age 13 research clinic, were also analysed. At both time points, height was measured using a Harpenden stadiometer (Holtain Limited, Wales) and weight using a Tanita Body Fat Analyser.

Cortical bone mineral content (BMC_C_), cortical bone mineral density (BMD_C_) and cortical bone area (BA_C_), were measured on a single slice at the mid tibia using the Stratec XCT2000L (Germany). Periosteal circumference (PC), endosteal circumference (EC) and cortical thickness (CT) were derived using a circular ring model. A threshold routine was used for defining cortical bone, which specified a voxel with a density >650 mg/cm^3^ as cortical bone. Of the 4500 pQCT scans obtained in ALSPAC 89 were rejected as being of insufficient quality. The coefficients of variation (CVs) based on 139 ALSPAC subjects scanned a mean of 31 days apart, were 2.7%, 1.3% and 2.9% for BMC_C_, BMD_C_, BA_C_, respectively.

Total body BMD and BMD of the left femoral neck were measured using a Lunar Prodigy scanner, for which CVs were 1.0% (146 subjects) and 1.7% (166 subjects) respectively.

#### Discovery set genotyping

1760 ALSPAC individuals were genotyped using the Illumina HumanHap317K SNP chip. Markers with <1% minor allele frequency, >5% missing genotypes or which failed an exact test of Hardy-Weinberg equilibrium (HWE) (p<1×10^−7^) were excluded from further analysis. We also excluded any individuals who did not cluster with the CEU individuals in multidimensional scaling analysis, who had >5% missing data, heterozygosity of >36.4% or <34.3% and a male who scored heterozygous at many X chromosome loci. After data cleaning we were left with 1518 individuals (999 with pQCT data) and 315,807 SNPs. We carried out imputation to HapMap release 22 using Mach 1.0, Markov Chain Haplotyping [16].

#### Replication set genotyping

Genotyping (of the SNPs with p<1×10^−5^ in the GWAS meta-analysis) was carried out on the entire ALSPAC cohort for whom DNA was available (10121 individuals) by KBioscience (http://www.kbioscience.co.uk), who employ a novel form of competitive allele specific PCR (KASPar) for genotyping. Those individuals who were included in the ALSPAC discovery analysis as well as those of non-white reported ethnicity, with >10% missing genotypes and with siblings in the cohort were excluded from the analysis. For each SNP there were between 2753 and 2789 individuals with both pQCT and genotype data. All but one SNP (rs9541712) were successfully genotyped. All were in HWE.

#### Measurement of free RANKL levels

Soluble RANKL levels (sRANKL) were measured on fasting blood samples (which were obtained according to standard protocols, collected in heparin tubes, and stored at minus 80 degrees until further use), in 37 male individuals attending the age 15 research clinic, randomly selected after stratification by *RANKL* rs1021188 genotype. Measurements, which were performed blind, were carried out using the ampli-sRANKL enzyme-linked immunosorbent assay (ELISA) from Biomedica (Vienna, Austria) according to manufacturer's instructions, with the exception that 90µl of plasma was used per well. Measurement ranges, intra- and inter-assay coefficients of variation (CVs) were 0.02–2pmol/l, <9% and <6% respectively. Duplicate samples with a coefficient of variation of <20% were considered for further statistical analysis. In sample(s) where the recorded concentration was below the detection limit of 0.02 pmol/l, the latter value was entered in subsequent analyses.

### GOOD cohort

#### Participants

The Gothenburg Osteoporosis and Obesity Determinants (GOOD) study was initiated to determine both environmental and genetic factors involved in the regulation of bone and fat mass [17], [18]. Male study subjects were randomly identified in the greater Gothenburg area in Sweden using national population registers, contacted by telephone, and invited to participate. To be enrolled in the GOOD study, subjects had to be between 18 and 20 years of age. There were no other exclusion criteria, and 49% of the study candidates agreed to participate (n = 1068). The subjects of the GOOD cohort were also analysed after five years of follow-up, between 23 and 25 years of age. The GOOD study was approved by the local ethics committee at Gothenburg University. Written and oral informed consent was obtained from all study participants. Height was measured using a wall-mounted stadiometer, and weight was measured to the nearest 0.1 kg.

#### Bone measures

BMD_C_, BMD_C_ and BA_C_ were measured on a single tibial diaphyseal slice (at 25% of the bone length in the proximal direction of the distal end of the bone) using the Stratec XCT2000 (Germany). PC, EC and CT were derived using a circular ring model. A threshold routine was used for defining cortical bone, which specified a voxel with a density >710 mg/cm^3^ as cortical bone. Trabecular vBMD was measured using a scan through the distal metaphysis (at 4% of the bone length in the proximal direction of the distal end of the bone) of tibia. Tibia length was measured from the medial malleolus to the medial condyle of the tibia. The CVs were <1% for all pQCT measurements.

aBMD (g/cm^2^) of the whole body, femoral neck (of the left leg), and lumbar spine were assessed using the Lunar Prodigy DXA (GE Lunar, Madison, WI, USA). The CVs for the aBMD measurements ranged from 0.5% to 3%, depending on application.

#### Discovery set genotyping

Genotyping was performed with Illumina HumanHap610 arrays at the Genetic Laboratory, Department of Internal Medicine, Erasmus Medical Center, Rotterdam, the Netherlands. Genotypes were called using the BeadStudio calling algorithm. Genotypes from 935 individuals passed the sample quality control criteria [exclusion criteria: sample call rate <97.5%, gender discrepancy with genetic data from X-linked markers, excess autosomal heterozygosity >0.33 (∼FDR<0.1%), duplicates and/or first degree relatives identified using IBS probabilities (>97%), ethnic outliers (3 SD away from the population mean) using multi-dimensional scaling analysis with four principal components]. Across 22 duplicate samples, genotype concordance exceeded 99.9%. We carried out imputation to HapMap release 22 (after excluding SNPs with MAF<1%, SNP call rate <98% and HWE p value <1×10^−6^) using Mach 1.0, Markov Chain Haplotyping [16].

### MrOS Sweden cohort

#### Participants

The Osteoporotic Fractures in Men (MrOS) study is a multicenter, prospective study including older men in Sweden (3014), Hong Kong (≅2000), and the United States (≅6000). In the present study, associations between candidate polymorphism and skeletal parameters were investigated in the Swedish cohort (Table 1), which consists of three sub-cohorts from three different Swedish cities (n = 1005 in Malmö, n = 1010 in Göteborg, and n = 999 in Uppsala). Study subjects (men aged 69–81) were randomly identified using national population registers, contacted and asked to participate. To be eligible for the study, the subjects had to be able to walk without assistance, provide self-reported data, and sign an informed consent; there were no other exclusion criteria [19]. The study was approved by the ethics committees at the Universities of Gothenburg, Lund, and Uppsala. Informed consent was obtained from all study participants.

#### Bone measures

Validated pQCT analyses were available for the Gothenburg and Malmö cohorts. BMC_C_, BMD_C_ and BA_C_, were measured on a single tibial diaphyseal slice slice (at 38% of the bone length in the proximal direction of the distal end of the bone) using the Stratec XCT2000 (Germany). PC, EC and CT were derived using a circular ring model. A threshold routine was used for defining cortical bone, which specified a voxel with a density >710 mg/cm^3^ as cortical bone. Trabecular vBMD was measured using a scan through the distal metaphysis (at 4% of the bone length in the proximal direction of the distal end of the bone) of tibia. The CVs were <1% for all pQCT measurements. Adjustments for study centre were performed.

Total body areal BMD (aBMD, g/cm^2^), as well as aBMD of the femoral neck and lumbar spine (L_1_-L_4_) were assessed at baseline using the Lunar Prodigy dual energy X-ray absorptiometry (DXA) (n = 2004 from the Uppsala and Malmö cohorts; GE Lunar Corp., Madison, WI, USA) or Hologic QDR 4500/A-Delphi (n = 1010 from the Göteborg cohort; Hologic, Waltham, MA, USA). The CVs for the aBMD measurements ranged from 0.5% to 3%, depending on the application. To be able to use DXA measurements performed with equipment from two different manufacturers, a standardized BMD (sBMD) was calculated, as previously described [19]. Adjustments for study centre were performed.

#### Replication set genotyping

Genotyping (of the SNPs with p<1×10^−5^ in the GWAS meta-analysis) was carried out using matrix-assisted laser desorption ionization-time of flight mass spectrometry on the Sequenom MassARRAY platform (San Diego, CA, USA). The genotyping call rate was >97% and the SNP-s were in Hardy-Weinberg equilibrium.

### Statistical methods

#### Genome-wide meta-analysis and replication

The ALSPAC discovery set (n = 999) and GOOD (n = 935) contributed to the genome-wide meta-analysis. We analysed only those imputed SNPs which had a minor allele frequency of >0.01 and an r^2^ imputation quality score of >0.3 in both sets (n = 2,417,199). We carried out genome-wide association analyses for BMD_C_ using additive linear regression in Mach2QTL for both ALSPAC and GOOD (using GRIMP [20] for the GOOD analyses). We included age, sex, height and weight(ln) as covariates in ALSPAC, and age, height and weight(ln) as covariates in the male only GOOD cohort.

We carried out meta-analyses of the results from the two cohorts using two methods in METAL (www.sph.umich.edu/csg/abecasis/metal). In the p-value meta-analysis study-specific Z-statistics are calculated (which summarise the p-values and direction of effect) for each SNP's association. The Z-statistics are then summed across studies, using weights proportional to the square-root of each study's sample size, to provide a summary p-value for each association. In the inverse variance method standardized betas and standard errors from each study are combined using a fixed effect model which weights the studies using the inverse variance. We also carried out the meta-analyses with and without genomic control. The results using each method were very similar and the selection of SNPs was based on the p-value meta-analysis not adjusted for genomic control. Genome-wide significance was taken to be p<5×10^−8^.

We selected one SNP from each independent region that had a p<1×10^−5^ for replication in the ALSPAC replication cohort and MrOS Sweden. Additive linear regression analyses were carried out for the associations between these SNPs and BMD_C_ in PLINK [21] (ALSPAC) or in SPSS Statistics 17.0 (MrOS Sweden) using age, sex, height and weight(ln) as covariates. We calculated the combined p-value (for all four cohorts) using METAL (method described above).

#### Association between the RANKL SNP and other traits

For the RANKL SNP (rs1021188) we also tested for associations with other bone traits; BA_C_, BMC_C_, PC, EC and CT using the pQCT data and TB BMD, FN BMD and LS BMD in all cohorts (where the appropriate measures were available). The ALSPAC discovery and replication cohorts were combined and we also show the association results from GOOD from two time-points (the original GWAS time-point (19 years) and data from the five year follow-up visit (24 years). We carried out association analyses using additive linear regression in PLINK for ALSPAC and in SPSS Statistics 17.0 for GOOD and MrOS Sweden. We included age, sex, height and weight(ln) as covariates in the model in ALSPAC, and age, height and weight(ln) as covariates in the male only GOOD and MrOS Sweden cohorts. Pubertal stage was also included as a covariate in the FN BMD analyses of ALSPAC.

An interaction between sex and rs1021188 in association with BMD_C_ in the ALSPAC cohort was tested in PLINK, which tests the significance of the interaction term in the linear model.

We carried out meta-analyses using standardized betas and standard errors from each of the studies. This was carried out in METAL using the inverse-variance method described above.

sRANKL levels were transformed using the Rank-Based Inverse Normal Transformation. Linear regression was used to determine the relationship between sRANKL level and the addition of the minor rs1021188 allele.

## Supporting Information

Table S1 All SNPs with p<1×10^−5^ in the discovery meta-analysis GWAS for BMDC. Shaded rows are those SNPs taken forward to replication.
(0.24 MB DOC)
