# Supplementary material for: Genome-Wide Association Meta-Analysis of Cortical Bone Mineral Density Unravels Allelic Heterogeneity at the RANKL Locus and Potential Pleiotropic Effects on Bone
Source: PLoS Genet. 2010 Nov 18;6(11):e1001217. doi: 10.1371/journal.pgen.1001217 (PMC2987837; doi:10.1371/journal.pgen.1001217)
Supplement: Table S1 — All SNPs with p<1×10^−5^ in the discovery meta-analysis GWAS for BMDC. Shaded rows are those SNPs taken forward to replication. (0.24 MB DOC) [file pgen.1001217.s001.doc]

**Supplementary Table S1.** All SNPs with p<1x10^-5^ in the discovery meta-analysis GWAS for BMD_C_. Shaded rows are those SNPs taken forward to replication.

|  |  |  |  | ALSPAC discovery | | | |  | GOOD | | | |  | | Meta-Analysis | |
| --- | --- | --- | --- | --- | --- | --- | --- | --- | --- | --- | --- | --- | --- | --- | --- | --- |
| chr | SNP | position |  | coding allele | beta | se | P |  | coding allele | beta | se | P |  | | P | |
| 7 | rs6974080 | 109833952 |  | T | 5.42 | 1.70 | 0.001431 |  | T | 3.95 | 1.20 | 0.001028 | |  | | 4.78E-06 |
| 7 | rs6954034 | 109834001 |  | G | -5.53 | 1.70 | 0.001155 |  | A | 3.96 | 1.20 | 0.001016 | |  | | 3.83E-06 |
| 7 | rs211793 | 109858750 |  | T | 6.08 | 1.72 | 0.0003994 |  | T | 3.70 | 1.23 | 0.002648 | |  | | 3.58E-06 |
| 7 | rs211790 | 109860146 |  | T | 6.11 | 1.72 | 0.0003694 |  | T | 3.70 | 1.23 | 0.002629 | |  | | 3.31E-06 |
| 7 | rs211797 | 109865473 |  | G | -6.21 | 1.67 | 0.0001963 |  | C | 3.96 | 1.22 | 0.001145 | |  | | 7.91E-07 |
| 7 | rs169833 | 109865785 |  | G | 6.21 | 1.67 | 0.0001943 |  | G | 3.96 | 1.22 | 0.001141 | |  | | 7.81E-07 |
| 7 | rs211812 | 109870288 |  | G | -6.21 | 1.66 | 0.0001907 |  | C | 3.95 | 1.21 | 0.001124 | |  | | 7.56E-07 |
| 7 | rs211811 | 109870908 |  | G | -7.25 | 1.83 | 0.0000776 |  | A | 3.85 | 1.32 | 0.00342 | |  | | 1.09E-06 |
| 7 | rs211810 | 109875760 |  | T | 6.21 | 1.66 | 0.0001828 |  | T | 3.95 | 1.21 | 0.001063 | |  | | 6.87E-07 |
| 7 | rs211809 | 109875859 |  | G | 6.21 | 1.66 | 0.0001821 |  | G | 3.95 | 1.21 | 0.001063 | |  | | 6.84E-07 |
| 7 | rs211808 | 109876557 |  | C | -6.21 | 1.66 | 0.0001823 |  | A | 3.95 | 1.21 | 0.001063 | |  | | 6.85E-07 |
| 7 | rs211804 | 109878696 |  | C | -6.35 | 1.64 | 0.0001046 |  | A | 3.94 | 1.21 | 0.001102 | |  | | 4.25E-07 |
| 7 | rs211803 | 109878984 |  | T | 6.23 | 1.67 | 0.0001896 |  | T | 3.95 | 1.21 | 0.001079 | |  | | 7.21E-07 |
| 7 | rs402946 | 109882292 |  | T | 6.23 | 1.67 | 0.00019 |  | T | 3.95 | 1.21 | 0.001092 | |  | | 7.31E-07 |
| 7 | rs416050 | 109882447 |  | G | 6.23 | 1.67 | 0.0001899 |  | G | 3.91 | 1.21 | 0.001217 | |  | | 8.16E-07 |
| 7 | rs7795082 | 109883510 |  | G | 6.23 | 1.67 | 0.0001902 |  | G | 3.48 | 1.18 | 0.003199 | |  | | 2.23E-06 |
| 7 | rs396911 | 109883921 |  | G | -6.23 | 1.67 | 0.0001907 |  | A | 3.48 | 1.18 | 0.003199 | |  | | 2.23E-06 |
| 7 | rs367898 | 109884276 |  | G | -6.23 | 1.67 | 0.0001908 |  | C | 3.48 | 1.18 | 0.003199 | |  | | 2.23E-06 |
| 7 | rs442451 | 109888782 |  | G | -6.24 | 1.67 | 0.0001906 |  | A | 3.47 | 1.18 | 0.003224 | |  | | 2.25E-06 |
| 7 | rs398554 | 109888839 |  | G | 6.24 | 1.67 | 0.0001907 |  | G | 3.47 | 1.18 | 0.003234 | |  | | 2.26E-06 |
| 7 | rs409006 | 109891686 |  | G | -6.26 | 1.68 | 0.00019 |  | A | 3.47 | 1.18 | 0.003305 | |  | | 2.30E-06 |
| 7 | rs388129 | 109893743 |  | G | 6.27 | 1.68 | 0.0001893 |  | G | 3.47 | 1.18 | 0.003348 | |  | | 2.33E-06 |
| 7 | rs10226721 | 109894584 |  | G | 6.28 | 1.68 | 0.0001892 |  | G | 3.47 | 1.18 | 0.003371 | |  | | 2.34E-06 |
| 8 | rs16877095 | 109123844 |  | T | 6.04 | 1.70 | 0.0003758 |  | T | 3.22 | 1.14 | 0.004836 | |  | | 6.32E-06 |
| 8 | rs16877097 | 109124104 |  | T | -6.04 | 1.70 | 0.0003791 |  | C | 3.22 | 1.14 | 0.004836 | |  | | 6.37E-06 |
| 12 | rs4280044 | 76117369 |  | G | -5.95 | 1.50 | 0.0000729 |  | G | -2.42 | 1.02 | 0.01792 | |  | | 6.90E-06 |
| 12 | rs2369599 | 76117710 |  | T | 5.95 | 1.50 | 0.0000729 |  | C | -2.42 | 1.02 | 0.01812 | |  | | 6.99E-06 |
| 12 | rs884220 | 76117925 |  | T | -5.95 | 1.50 | 0.0000729 |  | T | -2.41 | 1.02 | 0.01833 | |  | | 7.09E-06 |
| 12 | rs6538057 | 76121194 |  | G | -5.95 | 1.50 | 0.0000729 |  | G | -2.40 | 1.02 | 0.01876 | |  | | 7.29E-06 |
| 13 | rs9525613 | 41816648 |  | T | -4.00 | 1.34 | 0.002867 |  | T | -3.14 | 0.95 | 0.0009627 | |  | | 9.07E-06 |
| 13 | rs749934 | 42006245 |  | G | -4.71 | 1.33 | 0.000385 |  | G | -2.93 | 0.87 | 0.0007783 | |  | | 1.02E-06 |
| 13 | rs17458078 | 42009354 |  | C | 4.57 | 1.27 | 0.0003376 |  | A | -2.89 | 0.87 | 0.000846 | |  | | 9.74E-07 |
| 13 | rs9533143 | 42009405 |  | G | -4.59 | 1.27 | 0.0003141 |  | G | -2.89 | 0.87 | 0.0008473 | |  | | 9.11E-07 |
| 13 | rs1021188 | 42014133 |  | T | 7.63 | 1.67 | 4.72E-06 |  | C | -6.00 | 1.25 | 1.51E-06 | |  | | 3.27E-11 |
| 13 | rs1021189 | 42014211 |  | T | -4.60 | 1.27 | 0.0002985 |  | T | -2.88 | 0.87 | 0.0008547 | |  | | 8.75E-07 |
| 13 | rs17596685 | 42015201 |  | T | -7.58 | 1.66 | 5.13E-06 |  | T | -5.82 | 1.23 | 2.33E-06 | |  | | 5.36E-11 |
| 13 | rs1853856 | 42015771 |  | T | -4.84 | 1.25 | 0.0001033 |  | T | -2.86 | 0.87 | 0.0009964 | |  | | 3.79E-07 |
| 13 | rs9533146 | 42015900 |  | G | -4.84 | 1.25 | 0.000102 |  | G | -2.86 | 0.87 | 0.001002 | |  | | 3.77E-07 |
| 13 | rs168312 | 42019760 |  | G | -7.49 | 1.65 | 5.56E-06 |  | G | -5.74 | 1.23 | 2.86E-06 | |  | | 7.07E-11 |
| 13 | rs9533147 | 42021925 |  | T | 4.69 | 1.27 | 0.0002081 |  | C | -2.86 | 0.86 | 0.0008971 | |  | | 6.53E-07 |
| 13 | rs6561058 | 42024173 |  | T | 7.49 | 1.65 | 5.56E-06 |  | C | -5.73 | 1.23 | 3.02E-06 | |  | | 7.44E-11 |
| 13 | rs9525638 | 42026577 |  | T | -4.92 | 1.28 | 0.0001215 |  | C | 2.79 | 0.86 | 0.001264 | |  | | 5.62E-07 |
| 13 | rs9525639 | 42030879 |  | C | -7.49 | 1.65 | 5.56E-06 |  | C | -5.72 | 1.23 | 3.09E-06 | |  | | 7.60E-11 |
| 13 | rs4531631 | 42033131 |  | G | 7.49 | 1.65 | 5.56E-06 |  | A | -5.72 | 1.23 | 3.09E-06 | |  | | 7.60E-11 |
| 13 | rs7999416 | 42034346 |  | G | -7.34 | 1.70 | 0.000015 |  | G | -5.75 | 1.25 | 3.96E-06 | |  | | 2.63E-10 |
| 13 | rs7324282 | 42035704 |  | C | 7.50 | 1.65 | 5.45E-06 |  | A | -5.73 | 1.23 | 3.06E-06 | |  | | 7.38E-11 |
| 13 | rs1325798 | 42037049 |  | T | 4.93 | 1.28 | 0.000118 |  | T | 2.79 | 0.87 | 0.001262 | |  | | 5.47E-07 |
| 13 | rs7996068 | 42037390 |  | G | -7.41 | 1.65 | 7.18E-06 |  | G | -5.74 | 1.23 | 3.05E-06 | |  | | 9.72E-11 |
| 13 | rs8000846 | 42037586 |  | G | -7.52 | 1.65 | 5.18E-06 |  | G | -5.74 | 1.23 | 2.98E-06 | |  | | 6.85E-11 |
| 13 | rs9533154 | 42038102 |  | T | 4.94 | 1.28 | 0.0001135 |  | T | 2.79 | 0.87 | 0.001262 | |  | | 5.27E-07 |
| 13 | rs12585014 | 42038559 |  | G | 7.53 | 1.65 | 5.04E-06 |  | A | -5.67 | 1.23 | 3.89E-06 | |  | | 8.58E-11 |
| 13 | rs17536328 | 42041029 |  | T | 4.94 | 1.28 | 0.0001109 |  | C | -2.71 | 0.86 | 0.001716 | |  | | 7.11E-07 |
| 13 | rs7988338 | 42042842 |  | G | 7.54 | 1.65 | 4.95E-06 |  | A | -5.60 | 1.23 | 5.16E-06 | |  | | 1.11E-10 |
| 13 | rs7325635 | 42043319 |  | G | -4.95 | 1.28 | 0.0001101 |  | A | 2.71 | 0.86 | 0.001716 | |  | | 7.07E-07 |
| 13 | rs9533156 | 42045671 |  | T | -5.15 | 1.25 | 0.0000388 |  | T | -2.70 | 0.87 | 0.001878 | |  | | 3.07E-07 |
| 13 | rs9525641 | 42046024 |  | T | -5.18 | 1.25 | 0.000035 |  | T | -2.70 | 0.87 | 0.001892 | |  | | 2.83E-07 |
| 13 | rs4942143 | 42052806 |  | G | 7.60 | 1.65 | 4.28E-06 |  | A | -5.60 | 1.23 | 5.35E-06 | |  | | 9.92E-11 |
| 13 | rs2277439 | 42053443 |  | G | -7.60 | 1.65 | 4.22E-06 |  | G | -5.60 | 1.23 | 5.39E-06 | |  | | 9.85E-11 |
| 13 | rs1022926 | 42056734 |  | G | 7.61 | 1.65 | 4.17E-06 |  | C | -5.60 | 1.23 | 5.43E-06 | |  | | 9.80E-11 |
| 13 | rs4941433 | 42057135 |  | T | -7.61 | 1.65 | 4.13E-06 |  | T | -5.60 | 1.23 | 5.48E-06 | |  | | 9.80E-11 |
| 13 | rs9525643 | 42057516 |  | T | -4.98 | 1.28 | 0.0000963 |  | C | 2.69 | 0.86 | 0.001842 | |  | | 6.75E-07 |
| 13 | rs9525644 | 42057804 |  | G | -5.20 | 1.25 | 0.0000328 |  | G | -2.69 | 0.87 | 0.002015 | |  | | 2.87E-07 |
| 13 | rs2324851 | 42058930 |  | G | 7.66 | 1.65 | 3.65E-06 |  | A | -5.59 | 1.23 | 5.75E-06 | |  | | 9.08E-11 |
| 13 | rs2148073 | 42061799 |  | G | -7.69 | 1.64 | 2.75E-06 |  | G | -5.39 | 1.23 | 0.0000118 | |  | | 1.40E-10 |
| 13 | rs4356365 | 42062171 |  | T | -7.70 | 1.64 | 2.72E-06 |  | T | -5.39 | 1.23 | 0.000012 | |  | | 1.40E-10 |
| 13 | rs8002449 | 42062541 |  | G | -7.70 | 1.64 | 2.69E-06 |  | G | -5.38 | 1.23 | 0.0000121 | |  | | 1.41E-10 |
| 13 | rs346588 | 42063468 |  | G | -7.71 | 1.64 | 2.64E-06 |  | G | -5.37 | 1.23 | 0.0000126 | |  | | 1.43E-10 |
| 13 | rs346589 | 42064284 |  | G | 7.72 | 1.64 | 2.56E-06 |  | A | -5.35 | 1.23 | 0.0000135 | |  | | 1.49E-10 |
| 13 | rs346590 | 42064295 |  | G | -7.72 | 1.64 | 2.54E-06 |  | G | -5.35 | 1.23 | 0.0000134 | |  | | 1.47E-10 |
| 13 | rs1038434 | 42065333 |  | T | -7.73 | 1.64 | 2.50E-06 |  | T | -5.35 | 1.23 | 0.0000134 | |  | | 1.45E-10 |
| 13 | rs346591 | 42065474 |  | T | -7.73 | 1.64 | 2.49E-06 |  | T | -5.35 | 1.23 | 0.0000134 | |  | | 1.45E-10 |
| 13 | rs346592 | 42065513 |  | G | -7.71 | 1.63 | 2.34E-06 |  | G | -5.34 | 1.20 | 9.19E-06 | |  | | 9.33E-11 |
| 13 | rs1054016 | 42080002 |  | T | 4.98 | 1.28 | 0.0000984 |  | T | 2.64 | 0.89 | 0.002813 | |  | | 1.08E-06 |
| 13 | rs4338693 | 42081683 |  | T | 8.11 | 2.05 | 0.0000747 |  | C | -4.30 | 1.60 | 0.007151 | |  | | 2.39E-06 |
| 13 | rs9533172 | 42083132 |  | G | -7.30 | 1.56 | 2.84E-06 |  | G | -4.91 | 1.13 | 0.0000127 | |  | | 1.55E-10 |
| 13 | rs17596972 | 42084005 |  | G | 8.11 | 2.05 | 0.0000747 |  | A | -4.27 | 1.60 | 0.007573 | |  | | 2.56E-06 |
| 13 | rs9533173 | 42084976 |  | T | 4.51 | 1.28 | 0.000409 |  | T | 2.82 | 0.86 | 0.001023 | |  | | 1.41E-06 |
| 13 | rs9525647 | 42087121 |  | T | 4.57 | 1.28 | 0.0003537 |  | C | -2.57 | 0.87 | 0.002984 | |  | | 3.62E-06 |
| 13 | rs9533177 | 42087225 |  | T | 4.57 | 1.28 | 0.0003528 |  | G | -2.60 | 0.87 | 0.002733 | |  | | 3.30E-06 |
| 13 | rs1325804 | 42103517 |  | T | 8.66 | 2.12 | 0.0000429 |  | A | -4.40 | 1.59 | 0.005608 | |  | | 1.14E-06 |
| 13 | rs17066364 | 44413989 |  | G | 11.56 | 4.75 | 0.0148 |  | C | -11.53 | 2.82 | 0.0000447 | |  | | 4.44E-06 |
| 13 | rs17066368 | 44422443 |  | T | 11.50 | 4.72 | 0.01495 |  | A | -11.35 | 2.78 | 0.0000456 | |  | | 4.56E-06 |
| 13 | rs11620113 | 44448796 |  | G | -11.45 | 4.71 | 0.01511 |  | G | -11.12 | 2.73 | 0.000047 | |  | | 4.73E-06 |
| 13 | rs17066376 | 44453062 |  | T | -11.46 | 4.71 | 0.01508 |  | T | -11.11 | 2.73 | 0.0000469 | |  | | 4.72E-06 |
| 13 | rs11616867 | 44464816 |  | G | 11.46 | 4.71 | 0.01507 |  | A | -11.11 | 2.73 | 0.0000471 | |  | | 4.72E-06 |
| 13 | rs17334045 | 44467862 |  | G | -5.75 | 2.85 | 0.04403 |  | G | -7.57 | 1.73 | 0.0000125 | |  | | 7.28E-06 |
| 13 | rs11619825 | 44472057 |  | G | -11.47 | 4.72 | 0.01502 |  | G | -11.10 | 2.73 | 0.0000469 | |  | | 4.69E-06 |
| 13 | rs3783149 | 44480765 |  | G | 11.47 | 4.72 | 0.01501 |  | A | -11.09 | 2.73 | 0.000047 | |  | | 4.70E-06 |
| 13 | rs11620104 | 44485765 |  | G | 11.47 | 4.72 | 0.01499 |  | A | -11.09 | 2.72 | 0.000047 | |  | | 4.69E-06 |
| 13 | rs11147961 | 44487515 |  | G | -11.47 | 4.72 | 0.01497 |  | G | -11.09 | 2.72 | 0.0000471 | |  | | 4.69E-06 |
| 13 | rs3783148 | 44487854 |  | G | 5.76 | 2.85 | 0.04361 |  | A | -7.57 | 1.73 | 0.0000124 | |  | | 7.15E-06 |
| 13 | rs11618756 | 44493975 |  | G | -11.47 | 4.72 | 0.01508 |  | G | -11.09 | 2.72 | 0.0000471 | |  | | 4.73E-06 |
| 13 | rs7995712 | 44494683 |  | G | 5.77 | 2.86 | 0.04342 |  | C | -7.57 | 1.73 | 0.0000124 | |  | | 7.11E-06 |
| 13 | rs7995392 | 44494725 |  | T | -11.46 | 4.72 | 0.01513 |  | T | -11.09 | 2.72 | 0.0000471 | |  | | 4.75E-06 |
| 13 | rs7335692 | 44498715 |  | G | 11.46 | 4.72 | 0.01518 |  | C | -11.09 | 2.72 | 0.0000471 | |  | | 4.77E-06 |
| 13 | rs11618657 | 44498908 |  | T | -11.46 | 4.72 | 0.01522 |  | T | -11.09 | 2.72 | 0.0000471 | |  | | 4.78E-06 |
| 13 | rs11841716 | 44502491 |  | T | 11.45 | 4.72 | 0.01534 |  | G | -11.09 | 2.72 | 0.0000471 | |  | | 4.83E-06 |
| 13 | rs3949337 | 44502889 |  | T | 11.44 | 4.72 | 0.01537 |  | C | -11.10 | 2.73 | 0.0000469 | |  | | 4.82E-06 |
| 13 | rs11840919 | 44509646 |  | G | -11.44 | 4.72 | 0.01547 |  | G | -11.11 | 2.73 | 0.0000467 | |  | | 4.86E-06 |
| 13 | rs7332969 | 44510903 |  | G | -11.35 | 4.74 | 0.01669 |  | G | -11.12 | 2.73 | 0.0000467 | |  | | 5.33E-06 |
| 13 | rs7986584 | 44512775 |  | G | -11.34 | 4.74 | 0.0167 |  | G | -11.12 | 2.73 | 0.0000467 | |  | | 5.33E-06 |
| 13 | rs7324584 | 44514911 |  | G | -11.41 | 4.75 | 0.01638 |  | G | -11.17 | 2.74 | 0.0000456 | |  | | 5.11E-06 |
| 13 | rs9541712 | 68481740 |  | C | -2.92 | 1.33 | 0.02746 |  | A | 4.18 | 0.98 | 0.0000214 | |  | | 5.64E-06 |
| 13 | rs12869774 | 97898158 |  | G | 9.87 | 2.55 | 0.0001105 |  | A | -5.58 | 1.80 | 0.001916 | |  | | 7.96E-07 |
| 13 | rs7338502 | 97948011 |  | T | -12.05 | 2.88 | 0.0000284 |  | T | -8.34 | 2.20 | 0.0001466 | |  | | 1.62E-08 |
| 13 | rs12583333 | 97969327 |  | T | -12.07 | 2.88 | 0.0000274 |  | T | -8.29 | 2.20 | 0.0001618 | |  | | 1.73E-08 |
| 13 | rs7323905 | 97980694 |  | T | 11.84 | 3.55 | 0.0008673 |  | G | -8.43 | 2.57 | 0.001048 | |  | | 2.98E-06 |
| 18 | rs11875173 | 63006929 |  | T | -3.79 | 1.44 | 0.008599 |  | C | 3.80 | 1.03 | 0.0002369 | |  | | 8.81E-06 |
| 19 | rs8107232 | 56540636 |  | G | 3.53 | 1.30 | 0.00653 |  | G | 3.32 | 0.92 | 0.0003029 | |  | | 7.94E-06 |
| 19 | rs8107235 | 56540646 |  | G | 3.55 | 1.29 | 0.005757 |  | G | 3.32 | 0.91 | 0.0002725 | |  | | 6.31E-06 |
| 19 | rs8102334 | 56543541 |  | G | -3.70 | 1.28 | 0.003932 |  | G | -3.39 | 0.92 | 0.0002078 | |  | | 3.29E-06 |
